# Supplementary material for: Retrovirus-Derived RTL9 Plays an Important Role in Innate Antifungal Immunity in the Eutherian Brain
Source: Int J Mol Sci. 2023 Oct 4;24(19):14884. doi: 10.3390/ijms241914884 (PMC10573853; doi:10.3390/ijms241914884)
Supplement: Supplementary file 1 [file ijms-24-14884-s001.zip › ijms-2587672-supplementary.pdf]

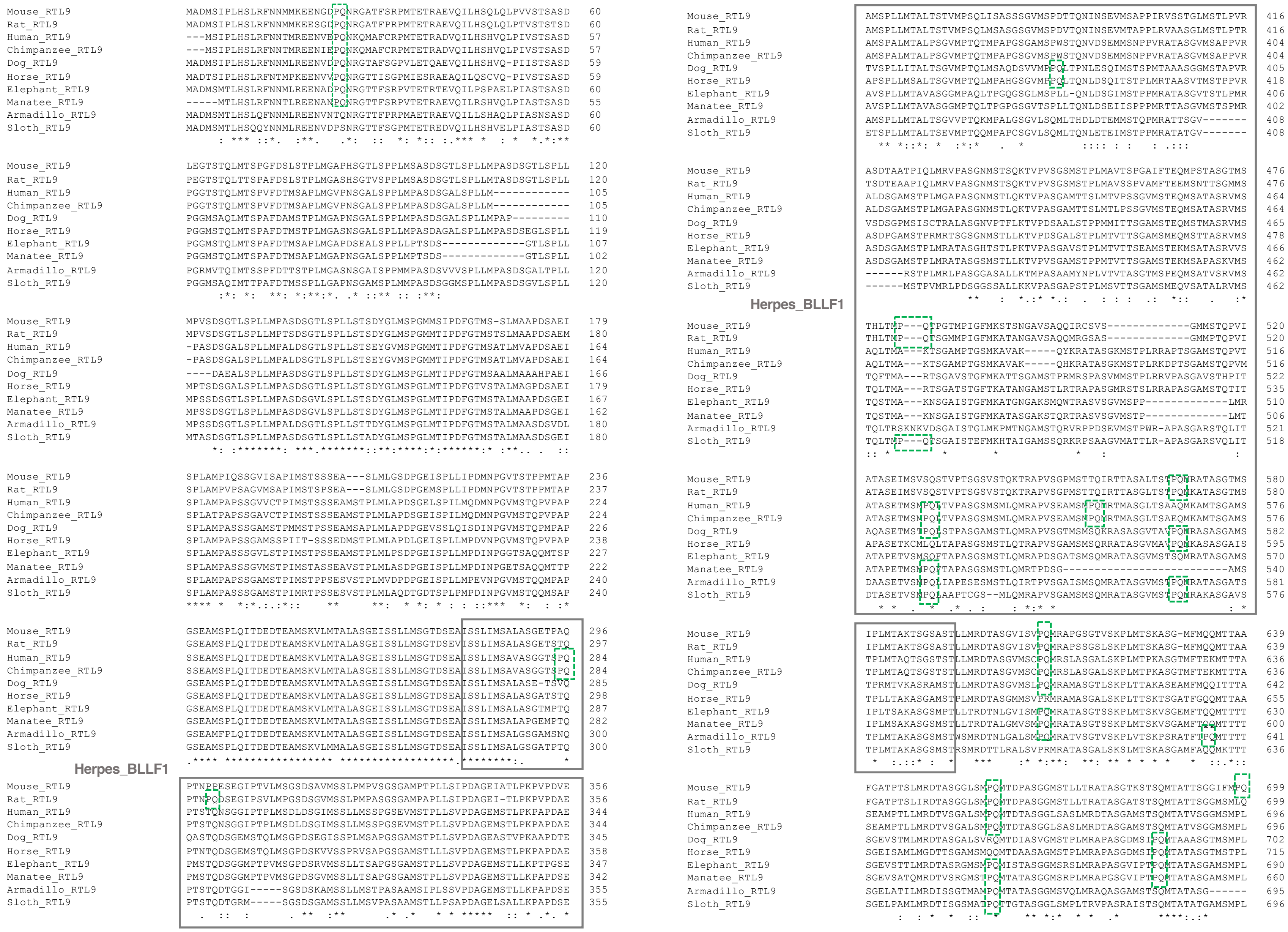

Fig. S1-1

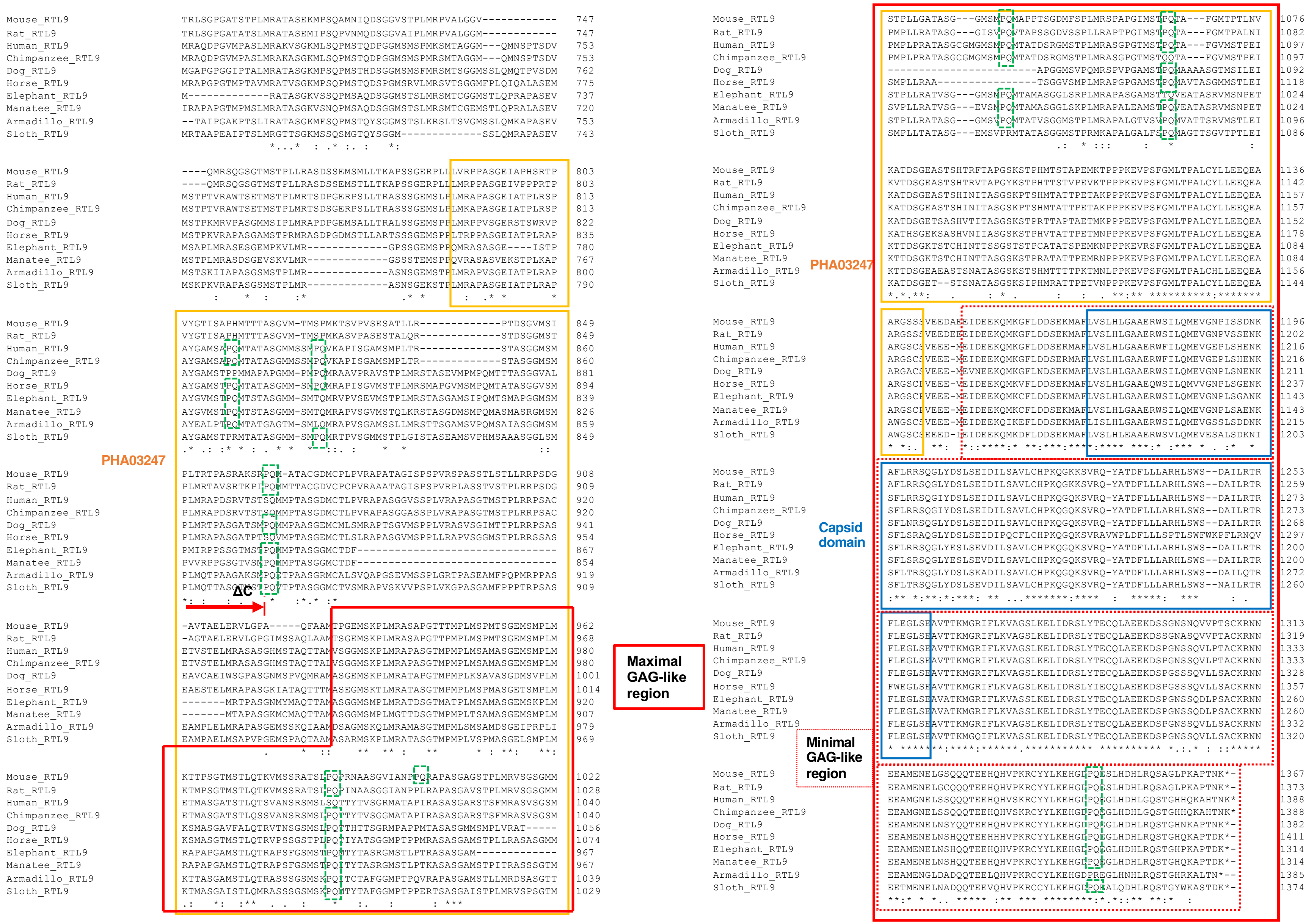
PHA03247
ΔC
Maximal GAG-like region
PHA03247
Capsid domain
Minimal GAG-like region

Fig. S1-2

Fig. S1 Comparison of the RTL9 protein in the 10 representative eutherian species

Multiple alignment of the RTL9 protein sequences in 10 eutherian species: the mouse, rat, human, chimpanzee, dog, horse, elephant, manatee, armadillo and sloth. The identical amino acids in the 10 species are depicted with an asterisk (*) below the alignment. Three regions corresponding to the herpes BLLF1 super family, PHA03247 super family and capsid domain of sushi-ichi GAG (Fig. 1A) are shown as gray, orange and blue boxes, respectively. Maximal and minimal GAG-like regions are shown as red boxes and red dashed boxes, respectively. The end of RTL9ΔC, just before the maximal GAG-like region, is indicated by a red arrow with a short vertical line on its head. Details are provided in the Comparative Genome analysis in the Materials and Methods section.

Conserved domains on [lcl|NM_001040434.2]

View Concise Results

NM_001040434.2 Mus musculus retrotransposon Gag like 9 (Rtl9), mRNA

Graphical summary Zoom to residue level show extra options »

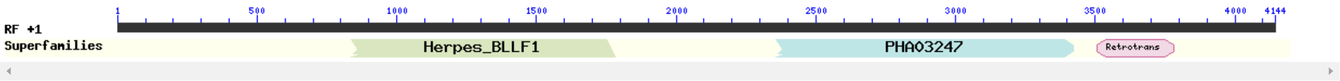

Search for similar domain architectures Refine search

List of domain hits

Name	Accession	Description	Interval	E-value
Retrotrans_gag super family	cl41948	Retrotransposon gag protein; Gag or Capsid-like proteins from LTR retrotransposons. There is a ...	3505-3780	7.31e-08

Retrotransposon gag protein; Gag or Capsid-like proteins from LTR retrotransposons. There is a central motif QGXXEXXXXXFXXLXXH that is common to Retroviridae gag-proteins, but is poorly conserved.

The actual alignment was detected with superfamily member pfam03732:

Pssm-ID: 455293 **Cd Length:** 97 **Bit Score:** 51.56 **E-value:** 7.31e-08

```
                             10        20        30        40        50        60        70        80
                     ....*....|....*....|....*....|....*....|....*....|....*....|....*....|....*....|
NM_001040434.2 1169 LVSLHL-GAAERW--SILQMEVGNPISSDN--KAFLRRsqgIYDSLSEIDILSAVLCHPKQGKKSVRQYATDFLLLAR-- 1241
Cdd:pfam03732     2 LAVHSLrGAALTWwkSLVARSIDAFDSWDEIkDAFLKR----FFPSIRKDLLRNELRSLRQGTESVREYVERFKRLARql 77

                             90       100
                     ....*....|....*....|
NM_001040434.2 1242 -HLSWSDAILRTRFLEGLSE 1260
Cdd:pfam03732    78 pHHGRDEEALISAFLRGLRP 97
```

Name	Accession	Description	Interval	E-value
PHA03247 super family	cl33720	large tegument protein UL36; Provisional	2359-3423	1.61e-06

large tegument protein UL36; Provisional

The actual alignment was detected with superfamily member PHA03247:

Pssm-ID: 223021 [Multi-domain] **Cd Length:** 3151 **Bit Score:** 53.02 **E-value:** 1.61e-06

```
                           10        20        30        40        50        60        70        80
                   ....*....|....*....|....*....|....*....|....*....|....*....|....*....|....*....|
NM_001040434.2  787 LVRPPASGEIAPHSRTPVYGTISAPHMTTTASGVMTMSPMKTSVPVSESATLLRPTDSGVMSIPLTRTPASRAKSRPQMA 866
Cdd:PHA03247   2698 LADPPPPPPTPEPAPHALVSATPLPPGPAAARQASPALPAAPAPPAVPAGPATPGGPARPARPPTTAGPPAPAPPAAPAA 2777

                           90       100       110       120       130       140       150       160
                   ....*....|....*....|....*....|....*....|....*....|....*....|....*....|....*....|
NM_001040434.2  867 TAcgdmcpIPVRAPATAGISPSPVRSPASStlstllrrPSDGAVTAELERVLGPAQFAAMTPGEMSKPLMRA--SAPGTT 944
Cdd:PHA03247   2778 GP-------PRRLTRPAVASLSESRESLPS--------PWDPADPPAAVLAPAAALPPAASPAGPLPPPTSAqpTAPPPP 2842

                          170       180       190       200       210       220       230       240
                   ....*....|....*....|....*....|....*....|....*....|....*....|....*....|....*....|
NM_001040434.2  945 TMPLMSPMTSGEMSMP---LMKTTPSGTMSTLQTKVMSSRATSLPQPrnaasgvianPPQRAPASGAGSTPLMRVSGSGM 1021
Cdd:PHA03247   2843 PGPPPPSLPLGGSVAPggdVRRRPPSRSPAAKPAAPARPPVRRLARP----------AVSRSTESFALPPDQPERPPQPQ 2912

                            250       260       270       280       290       300       310       320
                    ....*....|....*....|....*....|....*....|....*....|....*....|....*....|....*....|
NM_001040434.2 1022 MSTPLLGATASGGMSMPQMAPPTSGDMFSPLMRSPAPGIMSTPQtafGMTPTLNVKATDSGEASTSHTRFtapgskSTPH 1101
Cdd:PHA03247   2913 APPPPQPQPQPPPPPQPQPPPPPPPRPQPPLAPTTDPAGAGEPS---GAVPQPWLGALVPGRVAVPRFRV------PQPA 2983

                            330       340       350       360
                    ....*....|....*....|....*....|....*....|
NM_001040434.2 1102 MTSTAPEMKTPPPKEVPSFGMLTPALCYLLEEQEAARGSS 1141
Cdd:PHA03247   2984 PSREAPASSTPPLTGHSLSRVSSWASSLALHEETDPPPVS 3023
```

Fig. S2-1

Herpes_BLLF1 super family cl37540 Herpes virus major outer envelope glycoprotein (BLLF1); This family consists of the BLLF1 ... 838-1782 5.67e-03

Herpes virus major outer envelope glycoprotein (BLLF1); This family consists of the BLLF1 viral late glycoprotein, also termed gp350/220. It is the most abundantly expressed glycoprotein in the viral envelope of the Herpesviruses and is the major antigen responsible for stimulating the production of neutralising antibodies in vivo.

The actual alignment was detected with superfamily member pfam05109:

Pssm-ID: 282904 [Multi-domain] **Cd Length:** 886 **Bit Score:** 41.06 **E-value:** 5.67e-03

```
                            10        20        30        40        50        60        70        80
                    ....*....|....*....|....*....|....*....|....*....|....*....|....*....|....*....|
NM_001040434.2  280 ISSLIMSALASGETPAQPTNPPESEGIPTVL--MSGSDSAVMSSLPMPVSGSGAM--PTPLLSIPDAGEIA---TLPKPV 352
Cdd:pfam05109   473 VTSPTPAGTTSGASPVTPSPSPRDNGTESKApdMTSPTSAVTTPTPNATSPTPAVttPTPNATSPTLGKTSptsAVTTPT 552

                            90       100       110       120       130       140       150       160
                    ....*....|....*....|....*....|....*....|....*....|....*....|....*....|....*....|
NM_001040434.2  353 PDVEAMSPLLMTALTSTVMPSQLISASSSGVMSPdtTQNINSEVMSAPPIRVSST----GLMSTLPVRASDTAAtpiqlm 428
Cdd:pfam05109   553 PNATSPTPAVTTPTPNATIPTLGKTSPTSAVTTP--TPNATSPTVGETSPQANTTnhtlGGTSSTPVVTSPPKN------ 624

                           170       180       190       200       210       220       230       240
                    ....*....|....*....|....*....|....*....|....*....|....*....|....*....|....*....|
NM_001040434.2  429 rvpASGNMSTSQKTVPVSGSMSTPLmavtSPGAIFTEQMPSTASGTMSThltMPQTPGTMPIGFMKSTSNGAVSAQQIRC 508
Cdd:pfam05109   625 ---ATSAVTTGQHNITSSSTSSMSL----RPSSISETLSPSTSDNSTSH---MPLLTSAHPTGGENITQVTPASTSTHHV 694

                           250       260       270       280       290       300       310       320
                    ....*....|....*....|....*....|....*....|....*....|....*....|....*....|....*....|
NM_001040434.2  509 SVSGMMSTQPVIATASEIMSVSQSTVPTSGSVSTQKTRAPVSGPMSTTQIRTTASALTSTPQMRATASGTMSIPLMTAKT 588
Cdd:pfam05109   695 STSSPAPRPGTTSQASGPGNSSTSTKPGEVNVTKGTPPKNATSPQAPSGQKTAVPTVTSTGGKANSTTGGKHTTGHGART 774

                    ....*.
NM_001040434.2  589 SGSAST 594
Cdd:pfam05109   775 STEPTT 780
```

Fig. S2 The result of a conserved domain search using NCBI CDD

Three domains, the retrotransposon gag protein, large tegument protein UL36 and herpes virus major outer envelope glycoprotein (BLLF1), are listed in this order. The identical amino acids in the superfamily motifs and RTL9 are depicted in red.

Fig. S2-2

```
Mouse RTL9     1 ISSLIMSALASGETPAQPTNPPESEGIPTVL--MSGSDSAVMSSLPMPVSGSGAM--PTPLLSIPDAGEIA---TLPKPV     80
                 ::|...:...||.:|..|:..|...|..:..  |:...|||.:..|...|.:.|:  |||..:.|..|:.:   .:..|.
pfam05109      1 VTSPTPAGTTSGASPVTPSPSPRDNGTESKApdMTSPTSAVTTPTPNATSPTPAVttPTPNATSPTLGKTSptsAVTTPT     80

Mouse RTL9    81 PDVEAMSPLLMTALTSTVMPSQLISASSSGVMSPdtTQNINSEVMSAPPIRVSST----GLMSTLPVRASDTAAtpiqlm    160
                 |:..:.:|.:.|...:..:|:...::.:|.|.:|..|.|..|.:.......::|    |..|:.||..|....
pfam05109     81 PNATSPTPAVTTPTPNATIPTLGKTSPTSAVTTP--TPNATSPTVGETSPQANTTnhtlGGTSSTPVVTSPPKN------    160

Mouse RTL9   161 rvpASGNMSTSQKTVPVSGSMSTPLmavtSPGAIFTEQMPSTASGTMSThltMPQTPGTMPIGFMKSTSNGAVSAQQIRC    240
                 ...|:..::|.|..:..|.:.|..|.....|.:|.....|||:..:.|..  ||......|.|....|.....|......
pfam05109    161 ---ATSAVTTGQHNITSSSTSSMSL----RPSSISETLSPSTSDNSTSH---MPLLTSAHPTGGENITQVTPASTSTHHV    240

Mouse RTL9   241 SVSGMMSTQPVIATASEIMSVSQSTVPTSGSVSTQKTRAPVSGPMSTTQIRTTASALTSTPQMRATASGTMSIPLMTAKT    320
                 |.|.........:.||...:.|.||.|...:|:........:.|.:.:.:|....:|||.....:.:|........|:|
pfam05109    241 STSSPAPRPGTTSQASGPGNSSTSTKPGEVNVTKGTPPKNATSPQAPSGQKTAVPTVTSTGGKANSTTGGKHTTGHGART    320

Mouse RTL9   321 SGSAST    326
                 |...:|
pfam05109    321 STEPTT    326
```

Herpes_BLLF1 super family
identity 73/326 = 22.4%
Similarity 125/326 = 38.3%

Fig. S3 Pairwise alignment and identity between pfam05109 (BLLF1) and mouse RTL9

The N-terminal part of mouse RTL9 (280 aa – 594 aa) possessing homology to herpes BLLF1 super family motif was re-analyzed using EMBOS Water program and EMBOSS needle program as described in Materials and Methods. One and two dots represent similarities of amino acids, such as weekly and strong similar, respectively.

```
Mouse RTL9     1 LVRPPASGEIAPHSRTPVYGTISAPHMTTTASGVMTMSPMKTSVPVSESATLLRPTDSGVMSIPLTRTPASRAKSRPQMA     80
                 |..||........:...:......|.....|.......|...:.|...:........:.....|.|..|.:.|......|
PHA03247       1 LADPPPPPPTPEPAPHALVSATPLPPGPAAARQASPALPAAPAPPAVPAGPATPGGPARPARPPTTAGPPAPAPPAAPAA     80

Mouse RTL9    81 TAcgdmcplPVRAPATAGISPSPVRSPASStlstllrrPSDGAVTAELERVLGPAQFAAMTPGEMSKPLMRA--SAPGTT    160
                 ..       |.|....|..|.|..|....|        |.|.|...........|...|.:|.....|...|  :||...
PHA03247      81 GP-------PRRLTRPAVASLSESRESLPS--------PWDPADPPAAVLAPAAALPPAASPAGPLPPPTSAqpTAPPPP    160

Mouse RTL9   161 TMPLMSPMTSGEMSMP---LMKTTPSGTMSTLQTKVMSSRATSLPQPrnaasgvianPPQRAPASGAGSTPLMRVSGSGM    240
                 ..|....:..|....|   :.:..||.:.:.............|.:|          ...|:..|.|.............
PHA03247     161 PGPPPPSLPLGGSVAPggdVRRRPPSRSPAAKPAAPARPPVRRLARP----------AVSRSTESFALPPDQPERPPQPQ    240

Mouse RTL9   241 MSTPLLGATASGGMSMPQMAPPTSGDMFSPLMRSPAPGIMSTPQtafGMTPTLNVKATDSGEASTSHTRFtapgskSTPH    320
                 ...|............||..||.......||..:..|.....|.   |..|...:.|...|..:....|.      ..|.
PHA03247     241 APPPPQPQPQPPPPPQPQPPPPPPPRPQPPLAPTTDPAGAGEPS---GAVPQPWLGALVPGRVAVPRFRV------PQPA    320

Mouse RTL9   321 MTSTAPEMKTPPPKEVPSFGMLTPALCYLLEEQEAARGSS    360
                 .:..||...|||........:.:.|....|.|:......|
PHA03247     321 PSREAPASSTPPLTGHSLSRVSSWASSLALHEETDPPPVS    360
```

PHA03247 super family
identity 63/360 = 17.5%
similarity 85/360 = 23.6%

Fig. S4 Pairwise alignment and identity between PHA03247 family and mouse RTL9 protein

The middle of mouse RTL9 (787 aa –1141 aa) possessing homology to the PHA03247 super family motif was re-analyzed using the EMBOSS Water program and EMBOSS needle program. One and two dots represent similarities of amino acids, such as weekly and strong similar, respectively.

```
GAG            131  ELLFRHQPSRFVSDEAKVGFITSLLADKALSWAIAAVDLDPRLSSDYSAF   180
                    |:....|...|:.|..|:.|:.||....|..|:|..::...:|||..||
Mouse RTL9    1148  EIDEEKQMKGFLDDSEKMAFLVSLHLGAAERWSILQMEVGNPISSDNKAF  1197

GAG            181  RREFKAVFEHPTYGEDAASRLLALQQGSRSVAEYTLEFRILAAESRWGET   230
                    .|..:.:::..:..:..::.|...:||.:||.:|..:|.:||....|.:.
Mouse RTL9    1198  LRRSQGLYDSLSEIDILSAVLCHPKQGKKSVRQYATDFLLLARHLSWSDA  1247

GAG            231  ALRSAYRRGLSEAIKDLIVR---DRPSSLNELITLSLQMDERLRERRQER   277
                    .||:.:..|||||:...:.|   ....||.|||..||..:.:|.|.:.
Mouse RTL9    1248  ILRTRFLEGLSEAVTTKMGRIFLKVAGSLKELIDRSLYTECQLAEEKD--  1295

GAG            278  AQRAGGSTRQLSHRTSSAPDFSLTSTAAPPPHILLQSPAHPSPRVGEEPM   327
                       :.|::.|:      .|    ||                ..|..||.|
Mouse RTL9    1296  ---SSGNSNQV------VP----TS----------------CKRNNEEAM  1316

GAG            328  --QIGRSRLSRQEREQRLR-DQLCLYCGNNG---------HFIQACPVRPK   366
                     ::|    |:|:.|:... .:.|.|...:|        |..|:...: ||
Mouse RTL9    1317  ENELG----SQQQTEEHQHVPKRCYYLKEHGDPQESLHDHLRQSAGL-PK  1362

GAG            367  GPAHQ    371
                    .|.::
Mouse RTL9    1363  APTNK   1367
```

Red: Amino Acids encoded in the exon 2

Light blue: CCHC RNA-binding motif

Sushi-ichi GAG
identity 65/255 = 25.5%
similarity 110/255 = 43.1%

Fig. S5 Pairwise alignment and identity between sushi-ichi GAG and mouse RTL9 (1)

A conserved domain search using NCBI CDD indicated that the C-terminal portion of mouse RTL9 (1169 aa –1260 aa) possesses homology to the Capsid motif of sushi-ichi retrotransposon GAG. However, the regions before and after the Capsid motif (1148 aa-1168 aa and 1261 aa-1367 aa) also exhibit homology to GAG (**Minimal GAG-like region**). The AAs in *Rtl9* exon 2 are shown in red. The AAs of the CCHC motif of GAG are shown in light blue. One and two dots represent similarities of amino acids, such as weekly and strong similar, respectively.

```
GAG            6 TPGQ-------PGGPRTPTLP--SPLERRVEAHSAQLSSLQSELTKAFPT     46
                 |||:       ...|.|.|:|  ||:.      |.::|   ..|.|..|:
Mouse RTL9   927 TPGEMSKPLMRASAPGTTTMPLMSPMT------SGEMS---MPLMKTTPS    967

GAG           47 IQGEISELQSSSQTTSSTLSALSNQMSAMATVLASIIQK-----LGSDP-     90
                   |.:|.||  ::..||..::|....:|.:.|:|:..|:     .||.|
Mouse RTL9   968 --GTMSTLQ--TKVMSSRATSLPQPRNAASGVIANPPQRAPASGAGSTPL   1013

GAG           91 --------------GGAAPSEPSLP-LSPRAEPNLASP------------    113
                               |..|....|:| ::|....::.||
Mouse RTL9  1014 MRVSGSGMMSTPLLGATASGGMSMPQMAPPTSGDMFSPLMRSPAPGIMST   1063

GAG          114 --RVFG------------GDFDL---------GKGFLHQ-----------    129
                   ..||            |:...         .|...|.
Mouse RTL9  1064 PQTAFGMTPTLNVKATDSGEASTSHTRFTAPGSKSTPHMTSTAPEMKTPP   1113

GAG          130 --------------CELLFRHQPSR--------------------FVSDE    145
                               |.||...:.:|                    |:.|.
Mouse RTL9  1114 PKEVPSFGMLTPALCYLLEEQEAARGSSSVEEDAEEIDEEKQMKGFLDDS   1163

GAG          146 AKVGFITSLLADKALSWAIAAVDLDPRLSSDYSAFRREFKAVFEHPTYGE    195
                 .|:.|:.||....|..|:|..:::...:|||..||.|..:.:::..:..:
Mouse RTL9  1164 EKMAFLVSLHLGAAERWSILQMEVGNPISSDNKAFLRRSQGLYDSLSEID   1213

GAG          196 DAASRLLALQQGSRSVAEYTLEFRILAAESRWGETALRSAYRRGLSEAIK    245
                 ..::.|...:||.:||.:|..:|.:||....|.:..||:.:..|||||:.
Mouse RTL9  1214 ILSAVLCHPKQGKKSVRQYATDFLLLARHLSWSDAILRTRFLEGLSEAVT   1263

GAG          246 DLIVR---DRPSSLNELITLSLQMDERLRERRQERAQRAGGSTRQLSHRT    292
                 ..:.|   ....||.|||..||..:.:|.|.:.     :.|::.|:
Mouse RTL9  1264 TKMGRIFLKVAGSLKELIDRSLYTECQLAEEKD-----SSGNSNQV----   1304

GAG          293 SSAPDFSLTSTAAPPPHILLQSPAHPSPRVGEEPM--QIGRSRLSRQERE    340
                   .|    ||                ..|..||.|  ::|    |:|:.|
Mouse RTL9  1305 --VP----TS----------------CKRNNEEAMENELG----SQQQTE   1328

GAG          341 QRLR-DQLCLYCGNNG--------HFIQACPVRPKGPAHQ    371
                 :... .:.|.|...:|        |..|:..: ||.|.::
Mouse RTL9  1329 EHQHVPKRCYYLKEHGDPQESLHDHLRQSAGL-PKAPTNK   1367
```

Sushi-ichi GAG
identity 108/490 = 21.8%
similarity 175/490 = 35.7%

Fig. S6 Pairwise alignment and identity between sushi-ichi GAG and mouse RTL9 (2)

Entire sushi-ichi GAG exhibits homology to mouse RTL9 (927 aa-1357 aa, **Maximal GAG-like region**).

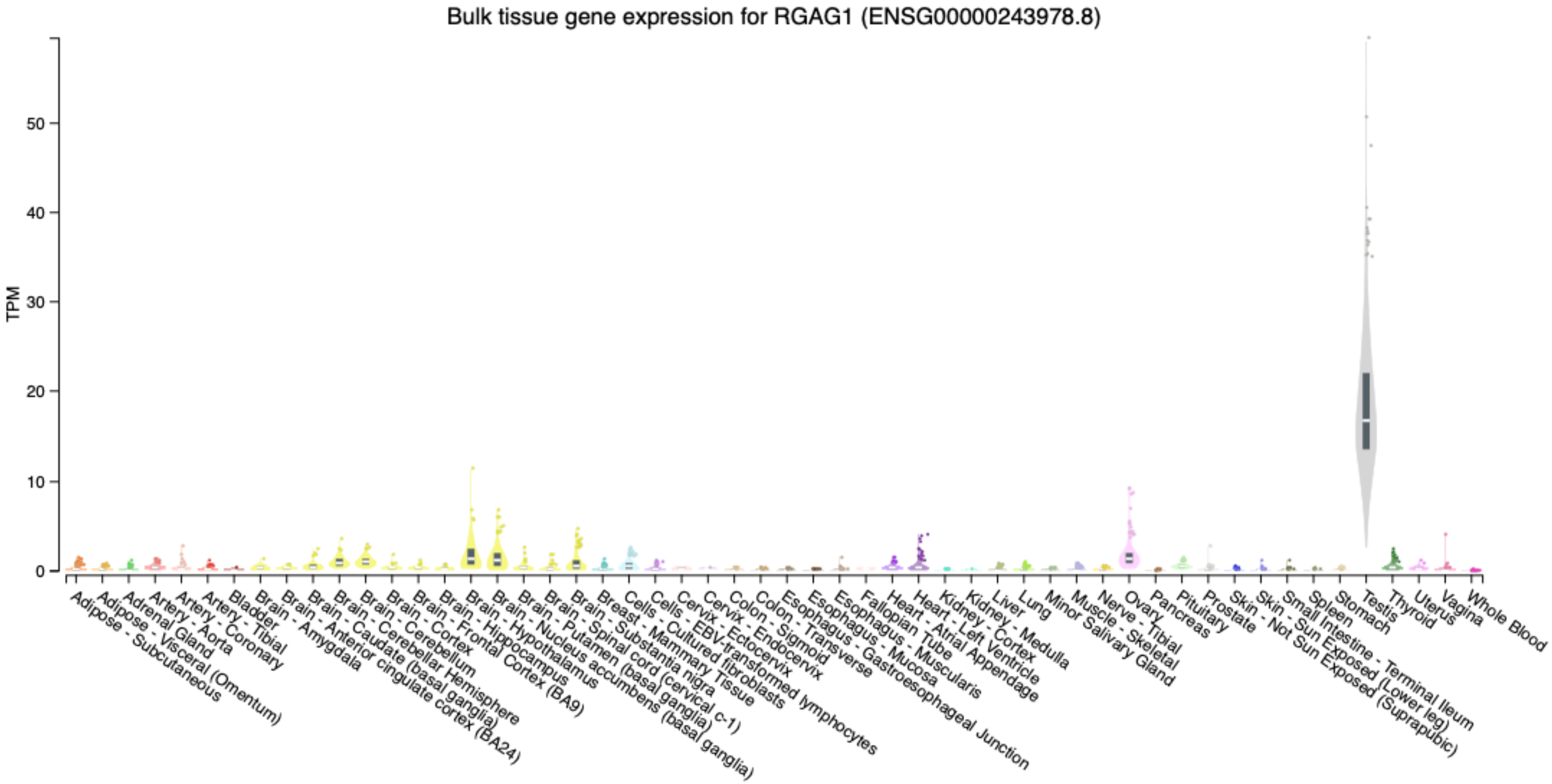

Fig. S7 ***RTL9*** **(*****RGAG1*****) expression in human tissues and organs**

See the original data at https://gtexportal.org/home/gene/RGAG1 (accessed on 2 June 2023)

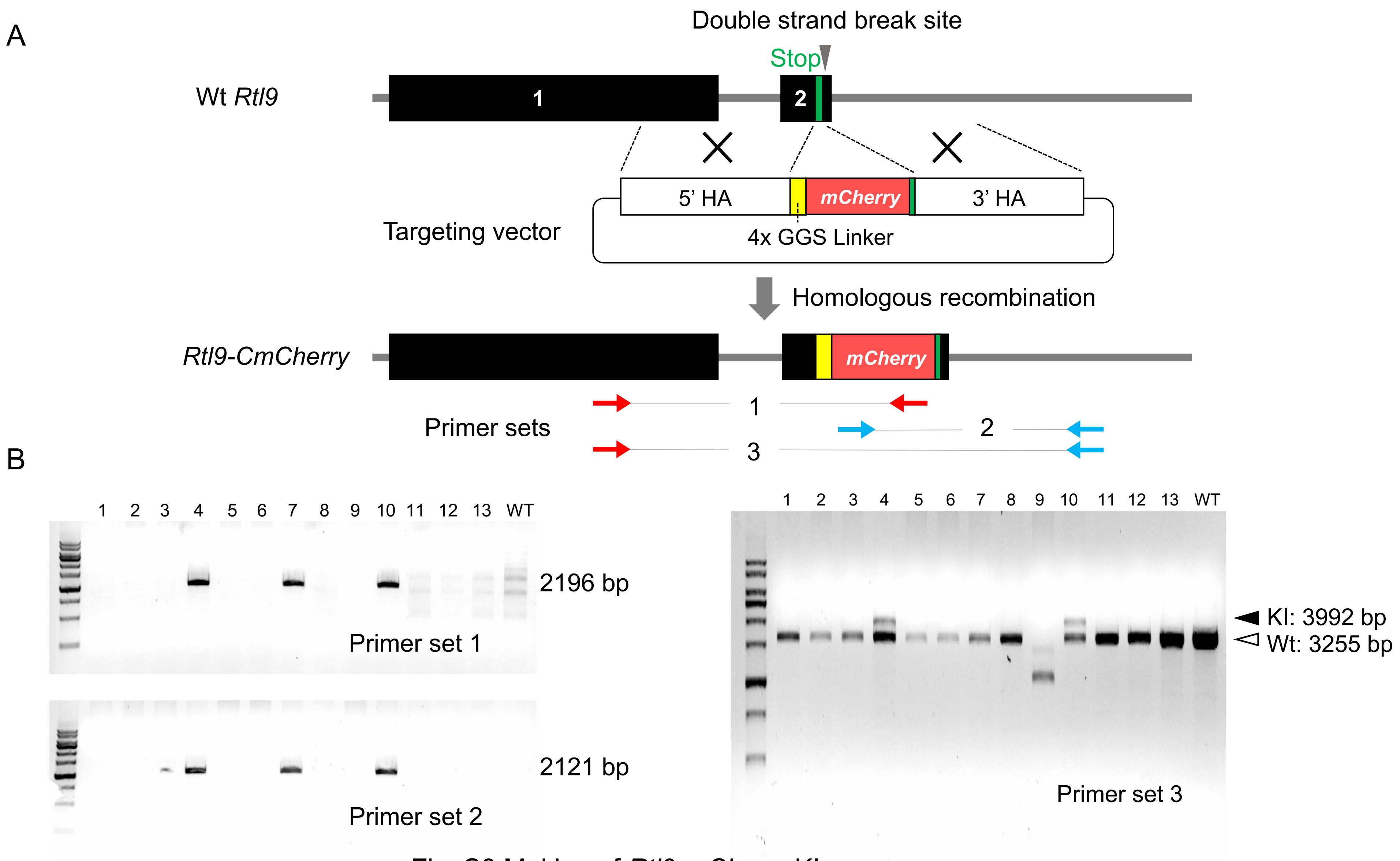

Fig. S8 Making of *Rtl9-mCherry* KI mouse

Fig. S8 Generation of the *Rtl9CmC* KI mouse

A) Schematic representation of the generation of the *Rtl9CmC* KI mouse. *Rtl9* exon 1 and 2 are numbered and shown in black. The stop codon is shown in green. The genomic locus near the stop codon in exon2 of *Rtl9* was cut out using the CRISPR/Cas system and homologous recombination was induced between the Wt *Rtl9* locus and a targeting vector containing 5' and 3' homology arms (5' HA and 3' HA), a 4x GGS linker and the mCherry coding sequence. The reconstructed *Rtl9CmC* has mCherry at the C-terminus. The position of the PCR primer sets for genotyping is indicated (red and light blue arrows). Cross symbols means homologous recombination. **B)** The results of genotyping 13 founder (F0) mice. Genomic PCR was performed using the primer sets 1 to 3 shown in A. The number (#) of F0 mice is indicated above the gel images. Wild type (WT) mouse genome was used as a control. Based on the genotyping PCR data, the offspring of F0 mouse #4 and #10 were used for further experiments.

PCR primer sets for genotyping of KI and KO mice

Mutant	Primer set name	Direction	Sequence
Rtl9-CmCherry	Primer set 1	F	GGAATGATGTCCACGCCACTA
		R	CTTCAGCTTCAGCCTCTGCT
	primer set 2	F	CCTGTCCCCTCAGTTCATGT
		R	CCTAGACTATTGGACCAGAGG
	Primer set 3	F	GGAATGATGTCCACGCCACTA
		R	CCTAGACTATTGGACCAGAGG
Rtl9-KO	Primer set 4	F	GAGAACACCAGCTTCTAGAGC
		R	GGGAGTTCAGAACCTCATACAC

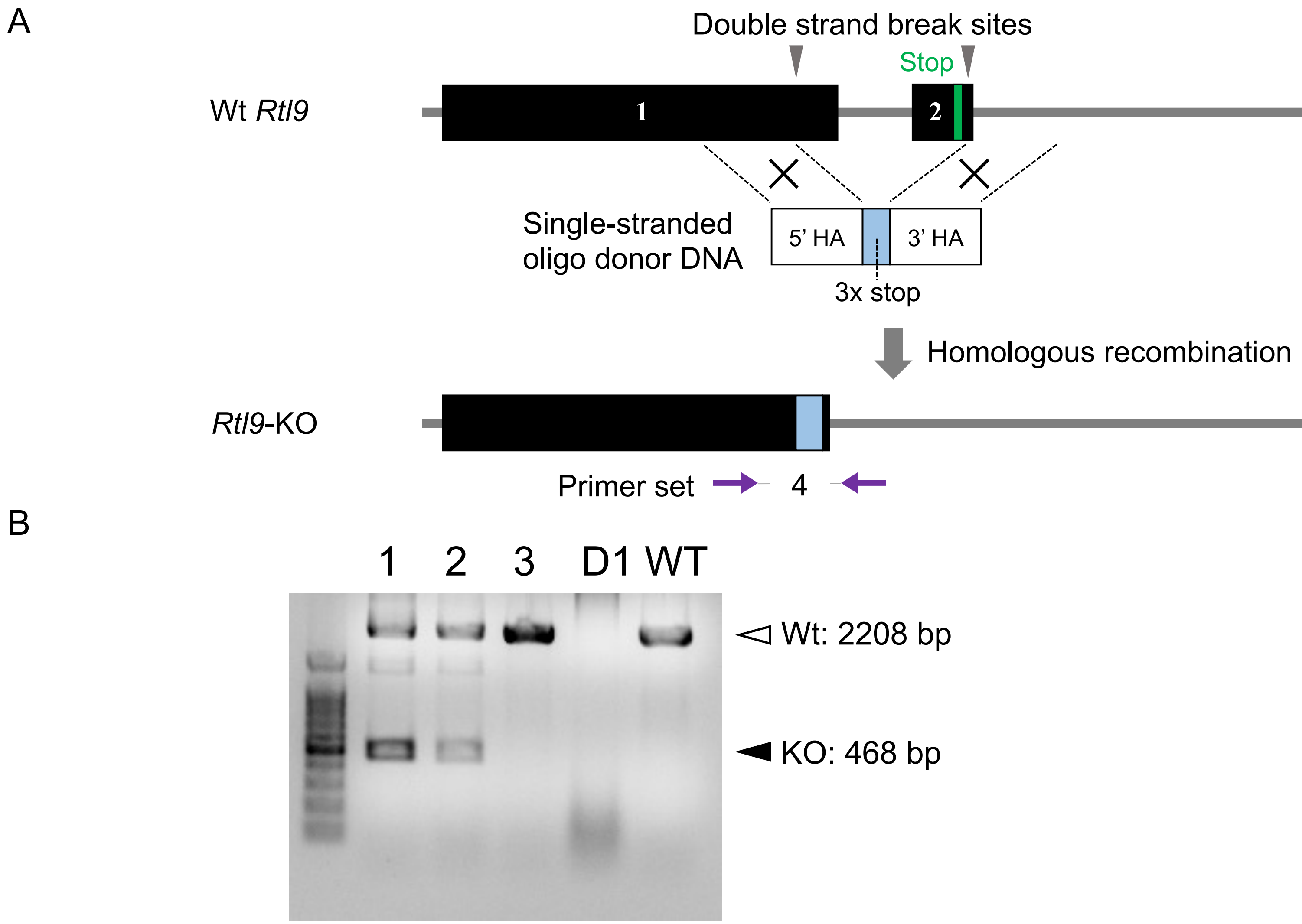

Fig. S9-1 Making of *Rtl9 KO* mouse

C

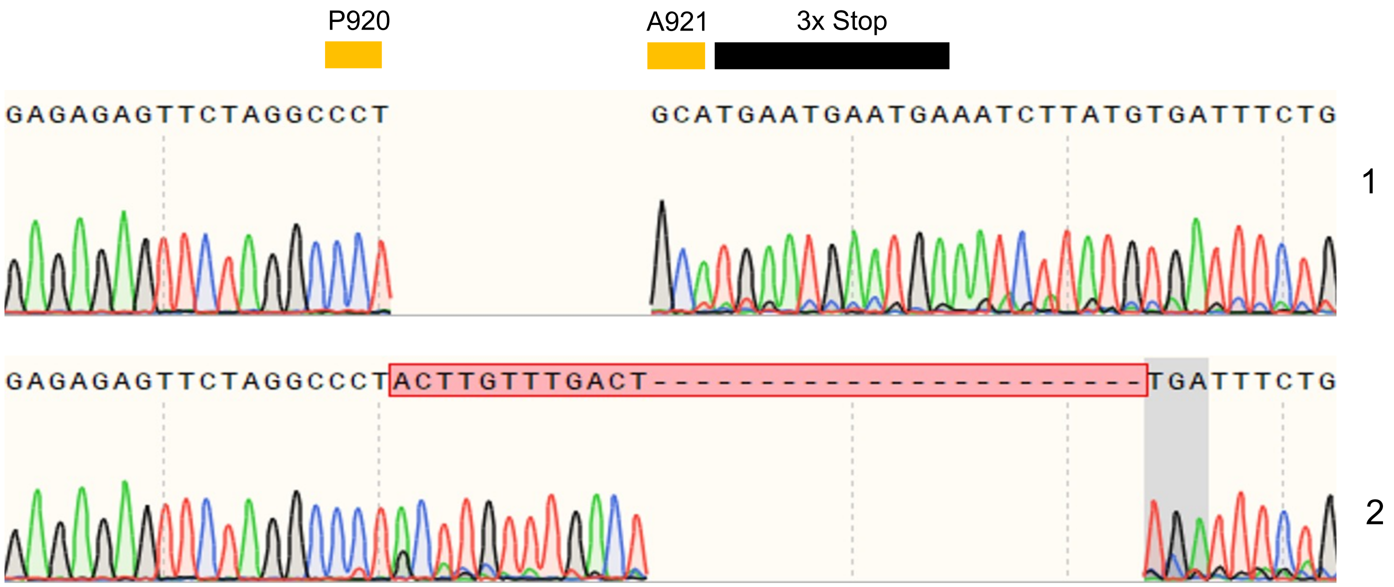

Fig. S9-2 Making of *Rtl9 KO* mouse

Fig. S9 Making of *Rtl9* KO mouse

A) Schematic representation of the generation of the *Rtl9* KO mouse. *Rtl9* exons 1 and 2 are numbered and shown in black. The stop codon is shown in green. The genomic locus encoding alanine 921 (A921) and downstream of the *Rtl9* stop codon was cut using the CRISPR/Cas system, and homologous recombination was induced between the Wt *Rtl9* locus and single-stranded oligo donor DNA containing a sequence of 3 stop codons (3x stop) and 5' and 3' homology arms (5' HA and 3' HA). In the reconstructed *Rtl9*-KO allele, the genomic region from glutamine 922 to the stop codon of *Rtl9* was deleted and replaced by a 3x stop. The position of the PCR primer set used for genotyping is indicated as purple arrows. **B)** Results of genotyping of 3 surviving and 1 dead (D1) F0 mice. Genomic PCR was performed with the primer set 4 shown in A. The number (#) of F0 mice, including D1, is indicated above the gel image. Wild type (WT) mouse genome was used as a control. **C)** Illustrated results of Sanger sequencing using the founder (F0) mice #1 and 2 shown in B. In F0 mouse #1 (top), a 3x stop was inserted at the expected genomic position just after A921 of *Rtl9*. In #2 (bottom), 12 extra bases (highlighted in red) were unexpectedly inserted just before a stop codon (highlighted in grey). Based on the Sanger sequencing data, the offspring of F0 mouse #1 were used for further experiments. The PCR primer sets for genotyping the KO mice are presented in the Fig. S6 legend.

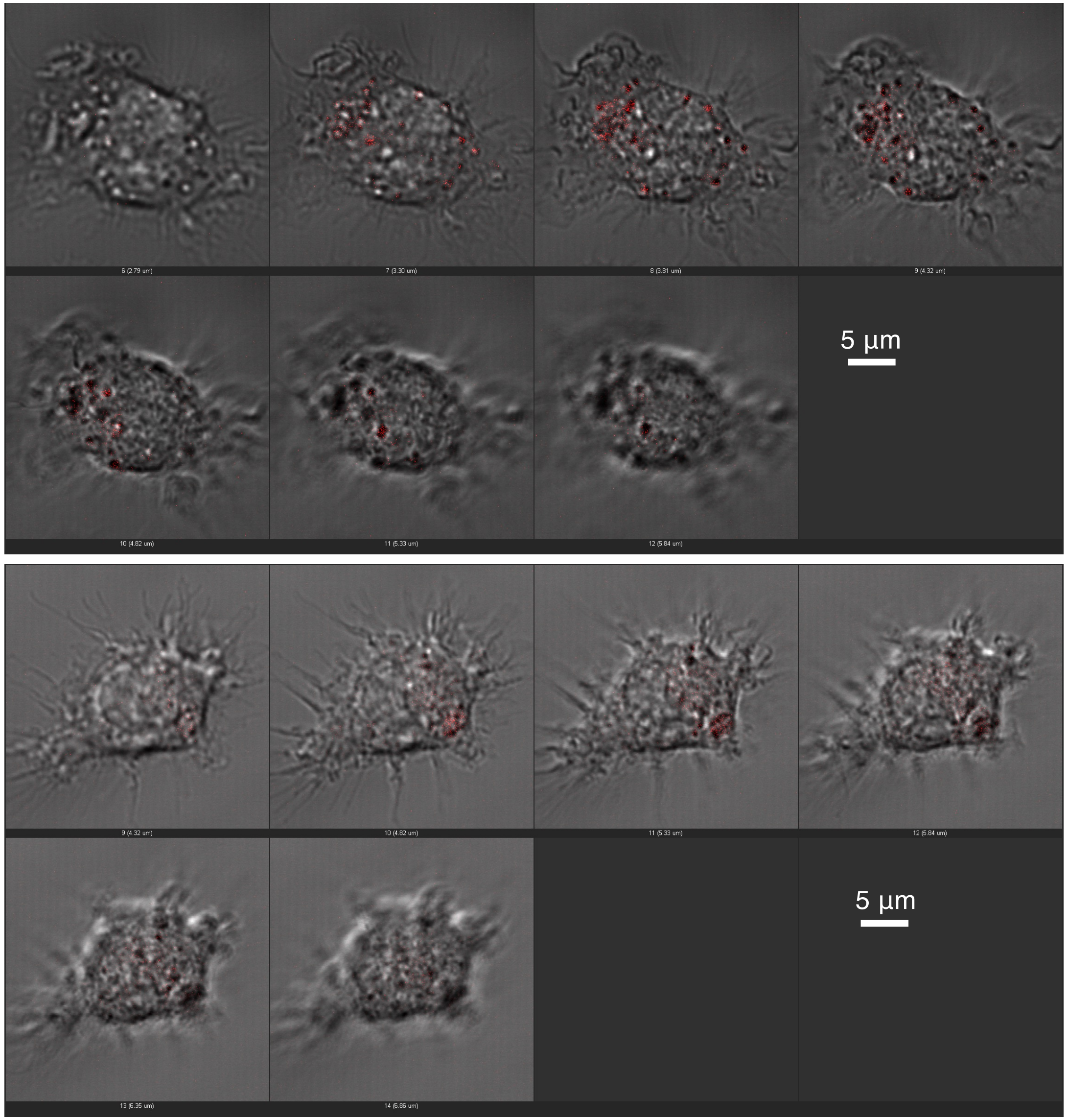

Fig. S10 mCherry signals in isolated microglia isolated from *Rtl9CmC* KI brain

Microglia were isolated from P0 neonatal brain and cultured for 10 days. The microglial cells were collected from the cultured Petri dishes (mixed glia cultures) by tapping (Lian et al., 2016; Irie et al., 2022).

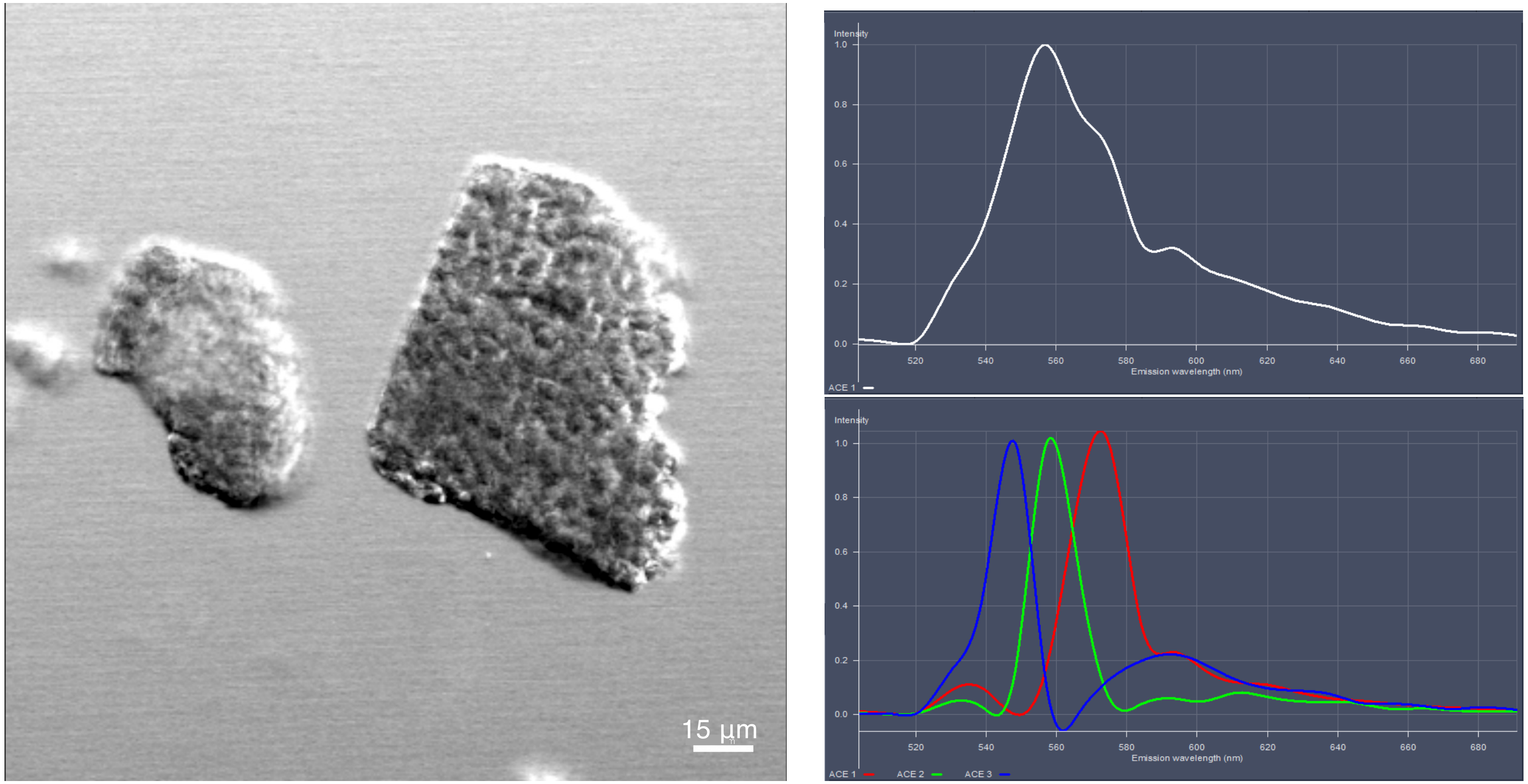

Fig. S11 The autofluorescence spectrum of Zymosan

Left: Transmission image. Right: Autofluorescence spectrum. Single (top) and/or the strongest signal (Maximum peak emission fluorescence wavelength: 570 nm) among 3 representative signals were used to detect zymosan.

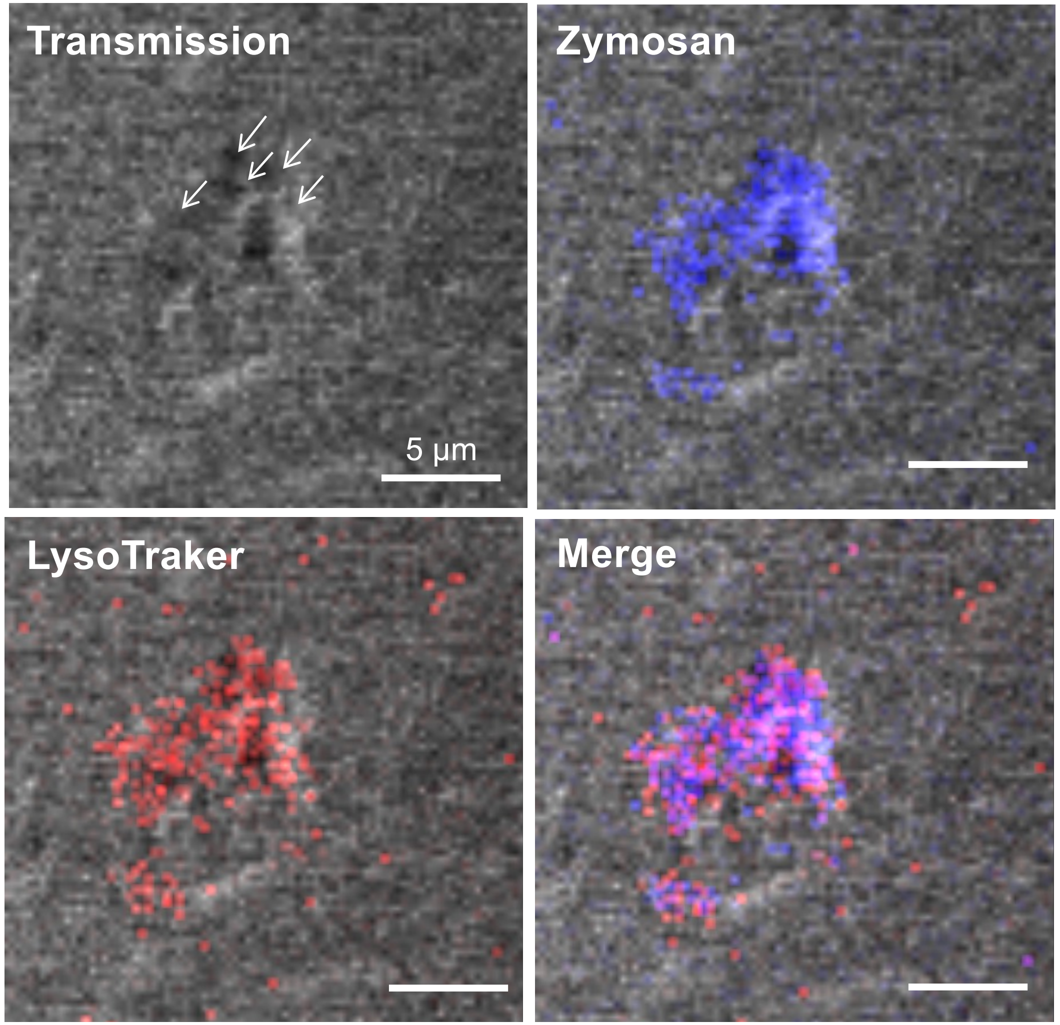

Fig. S12 Zymosan is visible in the transmission image

Zymosan incorporated in lysosomes in the *Rtl9* KO brain. Top, left: A transmission image. Arrows indicate zymosan aggregates. Top, right: zymosan autofluorescence (light blue). Bottom, left: The LysoTracker signal (red). Bottom, right: A merged image. Zymosan is distinguishable by its shape in the transmission image(s) in addition to its specific fluorescence signal. The same samples as in Fig. 3E were analyzed.

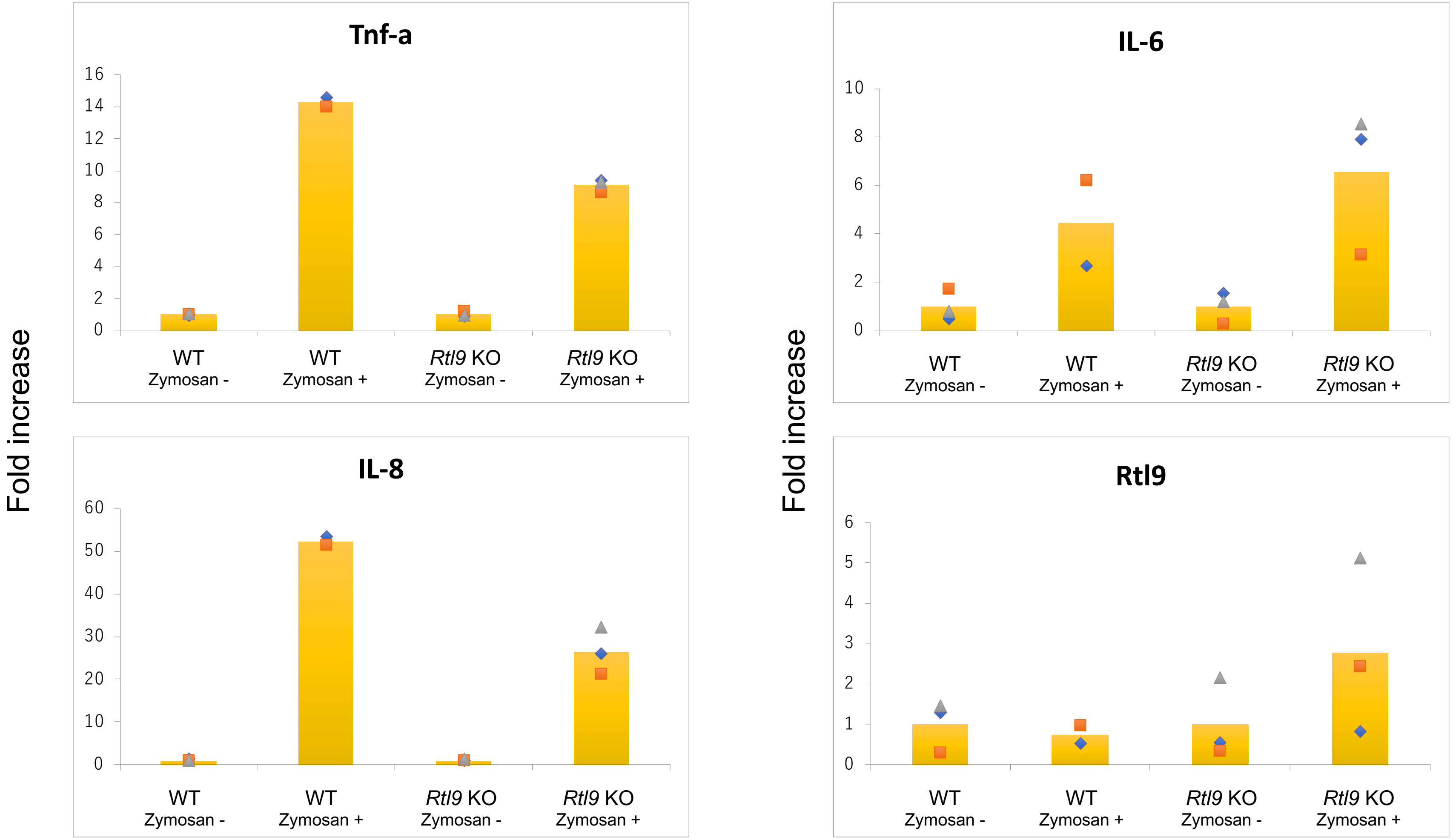

Fig. S13 Zymsan induces *Tnfa*, *Il6* and *Il8* expression in both WT and *Rtl9* KO microglia

Fig. S13 Zymosan induces *Tnfa*, *Il6* and *Il8* expression in both WT and *Rtl9* KO microglia

Tnfa, Il6, Il8 and *Rtl9* mRNA were analyzed at 0 min (no administration) and 30 min after zymosan administration to WT and *Rtl9* KO microglia (n=3 each, shown in different colors and shapes). The expression levels are averaged with non-administration samples as 1. *Rtl6* primers were designed for the 3’-UTR, and were therefore also present in the *Rtl9* KO microglia. It should be noted that the *Rtl9* mRNA itself increases upon zymosan administration in *Rtl9* KO microglia, presumably due to feedback regulation of the RTL9 protein.

The PCR primers used were as follows:

Tnfa, 5′-GACAAGGCTGCCCCGACTACG-3′ (forward) and 5′-CTTGGGGCAGGGGCTCTTGAC-3′ (reverse); *Il6*, 5′-AGTTGCCTTCTTGGGACTGA-3′(forward) and 5′-CCTCCGACTTGTGAAGTGGT-3′ (reverse); *Il8*: 5′-CTCTCAAGGGCGGTCAAAAAGTT-3′ (forward) and 5′-TCAGACAGCGAGGCACATCAGGTA-3′(reverse); *Rtl9*: 5'-TCACCTACATGCCTGTGACC-3' (forward) and 5'-CAACAACACCACATTGTTACGG-3' (reverse).
